# Supplementary material for: Identification of photoperiod-induced specific miRNAs in the adrenal glands of Sunite sheep (Ovis aries)
Source: Front Vet Sci. 2022 Jul 22;9:888207. doi: 10.3389/fvets.2022.888207 (PMC9354845; doi:10.3389/fvets.2022.888207)
Supplement: Supplementary file 1 [file Data_Sheet_1.ZIP › Supplementary file/miRDeep2 file.zip!/miRDeep2 file/Know_Structure/SP42A1/oar-mir-10b.pdf]

miRBase precursor	: oar-mir-10b
Total read count	: 685514
oar-miR-10b read count	: 683222
remaining reads	: 2292

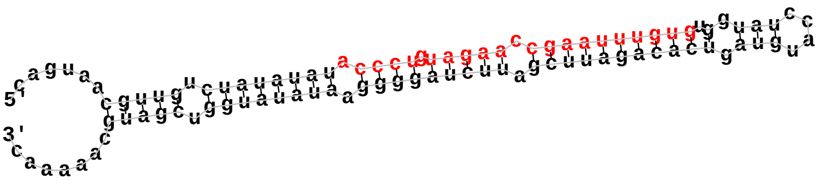

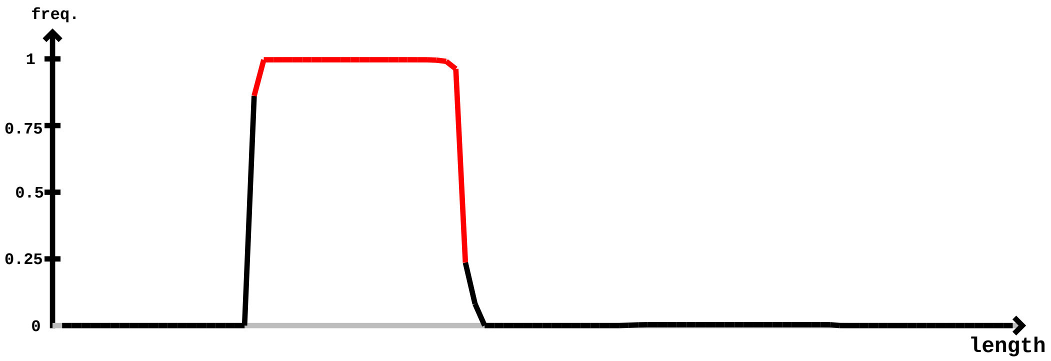


```
                                    oar-miR-10b
5'- caguaacguugucuauauauacccuguagaaccgaauuugugugguauccauguagucacagauucgauucuaggggaauauauggucgaugcaaaaac   -3'    exp
    ......(((((.((((((((.((((.(((((.((((((((((.(.(((....))).))))))))))).))))))))).)))))))).))))).......
```

sequence	reads	mm	sample
caguaacguugucuauauau...	1	0	seq
.................uauacccuguagaaccgaauuu..	1	0	seq
..................auacccuguagaaccgaauuugu..	17	0	seq
..................auacccuguagaaccgaauuugug...	2	0	seq
..................auacccuguagaaccgaauuugugu..	1	0	seq
...................uacccuguagaaccgaa...	6	0	seq
...................uacccuguagaaccgaau..	57	0	seq
...................uacccuguagaaccgaauu...	876	0	seq
...................uacccuguagaaccgaauuu..	2758	0	seq
...................uacccuguagaaccgaauuug...	17833	0	seq
...................uacccuguagaaccgaauuugu..	467283	0	seq
...................uacccuguagaaccgaauuugug...	78213	0	seq
...................uacccuguagaaccgaauuugugu..	23329	0	seq
...................uacccuguagaaccgaauuugugug...	19	0	seq
...................uacccuguagaaccgaauuuguguggg...	4	0	seq
...................uacccuguagaaccgaauuuguguggu...	1	0	seq
....................acccuguagaaccgaau..	10	0	seq
....................acccuguagaaccgaauu...	63	0	seq
....................acccuguagaaccgaCuu...	1	1	seq
....................acccuguagaaccgaCuuu..	1	1	seq
....................acccuguagaaccgaauuu..	292	0	seq
....................acccuguagaaccgaauuug...	1504	0	seq
....................acccuguagaaccgaauuugu..	30771	0	seq
....................acccuguagaaccgaauuugug...	27657	0	seq
....................acccuguagaaccgaauuugugu..	32375	0	seq
....................acccuguagaaccgaauuugugug...	65	0	seq
....................acccuguagaaccgaauuugugugg..	10	0	seq
....................acccuguagaaccgaauuuguguggu...	3	0	seq
.....................cccuguagaaccgaauuug...	1	0	seq
.....................cccuguagaaccgaauuugu..	15	0	seq
.....................cccuguagaaccgaauuugug...	8	0	seq
.....................cccuguagaaccgaauuugugu..	24	0	seq
......................ccuguagaaccgaauuugu..	9	0	seq
......................ccuguagaaccgaauuugug...	7	0	seq
......................ccuguagaaccgaauuugugu..	5	0	seq

oar-miR-10b

```
caguaacguugucuauauauacccuguagaaccgaauuugugugguauccauguagucacagauucgauucuaggggaauauauggucgaugcaaaaac
.......................cuguagaaccgaauuugu..........................................................   2     0     seq
.........................................................cacagauucgauucuagggga.....................   3     0     seq
.........................................................cacagauucgauucuaggggaa....................   5     0     seq
.........................................................cacagauucgauucuaggggaau...................   11    0     seq
..........................................................acagauucgauucuagggga.....................   23    0     seq
..........................................................acagauucgauucuaggggaa....................   44    0     seq
..........................................................acagauucgauucuaggggaau...................   765   0     seq
..........................................................acagauucgauucuaggggaaua..................   3     0     seq
..........................................................acagauucgauucuaggggaauau.................   1     0     seq
...........................................................cagauucgauucuagggg......................   1     0     seq
...........................................................cagauucgauucuagggga.....................   20    0     seq
...........................................................cagauucgauucuaggggaa....................   27    0     seq
...........................................................cagauucgauucuaggggaau...................   858   0     seq
...........................................................cagauucgauucuaggggaaua..................   16    0     seq
...........................................................cagauucgauucuaggggaauau.................   11    0     seq
............................................................agauucgauucuagggga.....................   6     0     seq
............................................................agauucgauucuaggggaa....................   8     0     seq
............................................................agauucgauucuaggggaau...................   434   0     seq
............................................................agauucgauucuaggggaaua..................   33    0     seq
............................................................agauucgauucuaggggaauau.................   20    0     seq
............................................................agauucgauucuaggggaauaua................   1     0     seq
```
